# Generalized modality responses in primary sensory neurons of awake mice during the development of neuropathic pain

**DOI:** 10.3389/fnins.2024.1368507

**Published:** 2024-04-16

**Authors:** Linlin Sun, Chao Chen, Xuwu Xiang, Shengyang Guo, Guang Yang

**Affiliations:** ^1^Department of Anesthesiology, Columbia University Medical Center, New York, NY, United States; ^2^Department of Physiology and Neuroscience, Skirball Institute of Biomolecular Medicine, New York University School of Medicine, New York, NY, United States; ^3^Department of Neurobiology, School of Basic Medicine, Peking University, Beijing, China

**Keywords:** primary sensory neurons, sensory modality, dorsal root ganglia, calcium imaging, neuropathic pain

## Abstract

**Introduction:**

Peripheral sensory neurons serve as the initial responders to the external environment. How these neurons react to different sensory stimuli, such as mechanical or thermal forces applied to the skin, remains unclear.

**Methods:**

Using *in vivo* two-photon Ca^2+^ imaging in the lumbar 4 dorsal root ganglion (DRG) of awake *Thy1.2*-GCaMP6s mice, we assessed neuronal responses to various mechanical (punctate or dynamic) and thermal forces (heat or cold) sequentially applied to the paw plantar surface.

**Results:**

Our data indicate that in normal awake male mice, approximately 14 and 38% of DRG neurons respond to either single or multiple modalities of stimulation. Anesthesia substantially reduces the number of responsive neurons but does not alter the ratio of cells exhibiting single-modal responses versus multi-modal responses. Following peripheral nerve injury, DRG cells exhibit a more than 5.1-fold increase in spontaneous neuronal activity and a 1.5-fold increase in sensory stimulus-evoked activity. As neuropathic pain resulting from nerve injury progresses, the polymodal nature of sensory neurons intensifies. The polymodal population increases from 39.1 to 56.9%, while the modality-specific population decreases from 14.7 to 5.0% within a period of 5 days.

**Discussion:**

Our study underscores polymodality as a significant characteristic of primary sensory neurons, which becomes more pronounced during the development of neuropathic pain.

## 1 Introduction

The somatosensory pathway used by the nervous system to perceive environmental information (e.g., mechanical and thermal forces acting on the skin) begins with neurons located within the dorsal root ganglia (DRG). These primary sensory neurons possess a pseudo-unipolar shape, with one axon extending into the skin and the other into the spinal cord. This structure allows them to serve as intermediaries between the periphery and the central nervous system. DRG sensory neurons display both anatomical and functional diversity, allowing them to be classified into distinct subpopulations based on several factors, including soma size (Martinez-Gutierrez and Castellanos, 2007), myelination of peripheral nerve fibers (Patzelt, 1953), molecular expression (Li et al., 2016), action potential velocity (Koerber et al., 1988), and their involvement in various sensory experiences such as touch, pain, and temperature (McMahon et al., 2013; Chisholm et al., 2018).

Despite ongoing efforts, studies on the sensory modalities of DRG sensory neurons have yielded inconclusive results. Prior research using electrophysiological recording and *in vitro* preparations suggests that most somatosensory neurons respond to multiple stimulus modalities, rendering them polymodal (Crain, 1956; Dichter and Fischbach, 1977; Djouhri and Lawson, 2004). Contradicting this perspective, recent studies propose that the majority of somatosensory neurons are modality-specific (i.e., respond exclusively to specific stimuli, such as noxious mechanical forces, heat or cold) (Abrahamsen et al., 2008; Lagerström et al., 2011; Han et al., 2013; Vrontou et al., 2013; Emery et al., 2016). The cause of these discrepancies remains unclear and may be attributed to differences in experimental models (e.g., *in vitro*, *ex vivo*, or *in vivo*) and recording methods. Additionally, the complexity is compounded by the fact that nerve injuries or insults to peripheral nerve endings of somatosensory neurons may unmask previously silent nociceptors (Perl, 1996; Zhang et al., 2022), induce changes in molecular phenotypes (Wang et al., 2011; Lisi et al., 2017), and even alter modality responses (Emery et al., 2016). Moreover, the modality responses of DRG neurons may be obscured by anesthetics, which can significantly influence neuronal activity. Therefore, there is a need for an animal preparation that enables the faithful recording of peripheral sensory stimulus-evoked physiological activity in primary sensory neurons while eliminating the confounding effects of general anesthesia.

We have recently developed an awake animal imaging technique that allows us to chronically visualize the mouse DRG at cellular and subcellular resolutions over days to weeks (Chen et al., 2019). Taking advantage of transgenic mice expressing a genetically encoded calcium indicator, GCaMP6s, in primary sensory neurons (Cichon et al., 2020), we performed *in vivo* Ca^2+^ imaging on the same population of DRG cells while subjecting them to various stimulus modalities, including punctate and dynamic mechanical force, as well as noxious heat and cold. In awake mice, approximately 52% of DRG neurons responded to peripheral sensory stimuli, with 74% of them displaying polymodal characteristics. When animals were anesthetized, the responsive cell population significantly decreased, but polymodality remained a primary feature of DRG neurons. Peripheral nerve injury intensified the polymodal feature of DRG sensory neurons. Within 5 days of the initiation of neuropathic pain, the population of modality-specific neurons decreased from 14.7 to 5.0%, while the polymodal population simultaneously increased from 39.1 to 56.9%. Our data suggest that polymodality is a significant feature of DRG sensory neurons in awake and anesthetized mice, both in the presence and absence of peripheral neuropathy.

## 2 Materials and methods

### 2.1 Mice

Transgenic mice expressing GCaMP6s in a subset of afferent neurons in the DRGs, specifically *Thy1*-GCaMP6 slow founder line 3, have been previously described (Cichon et al., 2020). The mice were generated at New York University School of Medicine. GCaMP6 slow coding regions were PCR amplified from GCaMP6s containing plasmids (Addgene, Cambridge, MA), a Kozak sequence was introduced, and the construct was verified by sequencing. GCaMP6s cDNA was then cloned into the SacII site of the mouse Thy1.2 promoter. The entire construct (the 6.5 kb of basic expression cassette and the 1.3 kb of transgene sequences) was purified after EcoRI and Pvul digestion and injected into fertilized B6SJLF1 mouse eggs at the Transgenic Mouse Lab facilities (Skirball Institute at NYU Langone Medical Center, NYULMC). Transgenic founders were backcrossed to C57BL/6J mice for the analysis of expression patterns. Primers for genotyping were: 5′- TCT GAG TGG CAA AGG ACC TTA GG -3′ (forward) 5′- TTA CGA CGT GAT GAG TCG ACC -3′ reverse. The *Thy1.2*-GCaMP6s line 3 mice were maintained at the NYULMC Skirball animal facility and Columbia University Medical Center animal facility.

In this study, male *Thy1.2*-GCaMP6s, aged 2 to 3 months, and wild-type C57BL/6 J mice were used. The mice were group-housed on a 12-h light–dark cycle under controlled temperature and humidity conditions, with ad libitum access to food and water. After the implantation of the DRG window, the mice were individually housed to reduce the risk of window damage. All experiments were conducted in accordance with protocols approved by the Institutional Animal Care and Use Committee (IACUC) at New York University (160905) and Columbia University (AAAW7462) as consistent with the National Institutes of Health (NIH) Guidelines for the Care and Use of Laboratory Animals to ensure minimal animal use and discomfort.

### 2.2 Surgical preparation for imaging awake, vertebrae-restrained mice

*In vivo* Ca^2+^ imaging was performed in vertebrae-restrained mice under awake or anesthetized conditions. The surgical procedure for preparing mice for awake animal imaging has been previously described (Chen et al., 2019). In brief, mice were deeply anesthetized through an intraperitoneal injection of 100 mg/kg ketamine and 15 mg/kg xylazine, and placed under a stereomicroscope (Leica MZ12.5; Leica Microsystems). To maintain eye moisture during the procedure, a hydrating lubricant was applied to both eyes. A 1-cm-long incision was made in the dorsal skin at the lumbar 3 (L3)–L5 level of the spine, and the muscles along the lateral aspects of L3–L5 were detached and then retracted with a custom-made metal sheet. To expose the left L4 DRG, we aligned the long bracket of the imaging device with the left side of L4–L5 vertebrae and short bracket with the right side, and then registered the two brackets with a locking screw. After trimming articular processes around the DRG, a thin layer of Kwik-Sil^®^ silicone elastomer and a round coverslip was placed on top. The coverslip was secured to the vertebrae mount by cyanoacrylate and dental acrylic. EMLA cream (Actavis pharma) was applied around the surgical site after window implantation once a day for 2 days. Step-by-step surgical procedures were detailed in our prior publication (Chen et al., 2019). Throughout the surgical procedure and recovery period, the animal’s body temperature was maintained at ~37°C.

### 2.3 *In vivo* Ca^2+^ imaging

*In vivo* imaging of the L4 DRG in awake mice was conducted more than 5–7 days post-surgery, as previously described (Chen et al., 2019). The mouse was vertebrae-fixed under the two-photon microscope. In brief, mice were placed inside a 2.9-cm-diameter transparent plastic cylinder securely affixed to a heavy metal base to minimize motion artifacts. Prior to imaging, the mice were habituated for at least 30 min.

For Ca^2+^ imaging of afferent sensory somata in the DRG, *Thy1.2*-GCaMP6s line 3 mice were used. The *in vivo* Ca^2+^ imaging experiments were carried out using a Bruker two-photon system equipped with a DeepSee Ti:sapphire laser (Spectra-Physics) tuned to 920 nm. Images were collected at frame rates of 2 Hz, with a resolution of 512 × 512 pixels, using a 25× objective (NA, 1.05) immersed in artificial cerebrospinal fluid, along with a 1× digital zoom. Image acquisition was performed using Bruker PrairieView software. To prevent tissue damage, the laser power reaching the DRG was restricted to ≤ 20 mW. Additionally, to avoid bleaching of fluorescent signals during imaging, we imposed a time limit of less than 10 min for each imaging session. Simultaneously, video recording of hindlimb movement was conducted, and only imaging sessions without hindlimb movement were included for spontaneous activity analysis.

### 2.4 Plantar stimulations

A pinch stimulus was applied by using obtuse-ended forceps in a way that covered about 1 mm^2^ region of the skin (Khandwala et al., 1997). Punctate mechanical stimulations were performed by using von Frey filaments with various forces on the plantar surface. Dynamic mechanical stimulations involved the use of a #5 paint brush, which was trimmed and used as previously described (Cheng et al., 2017). For thermal stimulations (heat and cold), a drop of hot (55°C) or cold (0°C) water (with a drop volume of ~300 μL) was applied via a 0.5 mL Pasteur pipette. The 55°C water was maintained by a dry bath. The 0°C water was a mixture of ice and water, and we delivered the water using the pipette.

### 2.5 Spared nerve injury

Spared sural nerve injury (SNI) of the sciatic nerve or sham operation was performed under sterile conditions (Cichon et al., 2018). In brief, adult mice (2–3 months old) were anesthetized through an intraperitoneal injection of 100 mg/kg ketamine and 15 mg/kg xylazine. A small incision was made in the left thigh to expose the sciatic nerve and its peripheral branches, which include the common peroneal, tibial, and sural nerves. An 8/0 nylon thread was carefully placed under the common peroneal and tibial nerves, followed by ligation and cutting, while the sural nerve remained untouched. Care was taken to avoid any stretching of the spared sural nerve. The layers of muscles and skin were then closed. For the sham surgery, the sciatic nerve was exposed but not ligated or cut.

### 2.6 Pain behavior tests

#### 2.6.1 Mechanical allodynia test

Mice were individually placed in clear acrylic boxes (10 cm × 7 cm × 7 cm), positioned on a mesh table, and allowed to habituate for at least 30 min before testing. Mechanical paw withdrawal thresholds were measured with the electronic von Frey anesthesiometer (IITC Life Science, catalog number: 2392) equipped with a #8 filament that precisely measures the minimum pressure at which paw withdrawal behavior occurs. The #8 von Frey tip, of suitable rigidity, was affixed to an electronic probe and applied with increasing pressure to the lateral plantar aspect of the hind paw (within the sural nerve skin territory). The anesthesiometer displayed the minimum applied force at which the mouse retracts from the von Frey tip. Three trials of paw withdrawal were recorded. This mechanical testing was conducted before surgery and at 3 and 5 days post-surgery, for both sham- and SNI-operated mice.

#### 2.6.2 Hot plate test

Mice were placed in Plexiglas chambers atop a hot plate (Ugo basile hot/cold plate). The plate was set for 55°C, allowing measurement of the time elapsed between the start of the paw touch and the paw lift. This time interval is defined as the paw withdrawal latency. Each trial was repeated three times at 10-min intervals. A cutoff time of 20 s was implemented to prevent tissue damage.

#### 2.6.3 Cold plate test

Paw withdrawal latencies to cold were measured using a cold plate (TE Technology, Inc., Traverse City, MI). The plate was equipped with a differential thermocouple thermometer (Harvard Apparatus, South Natick, MA) that provided a temperature precision of 0.1°C. Each animal was placed within an acrylic container (allowing for free movement) on the cold plate, which was set at 0°C. The time between placing the hind paw on the plate and the animal’s jumping response, with or without paw licking and flinching, was recorded as the paw withdrawal latency. Each trial was repeated three times at 10-min intervals. A cutoff time of 20 s was established to prevent potential damage to paw tissue.

#### 2.6.4 Conditioned place preference (CPP) test

The CPP test was conducted in accordance with established procedures (He et al., 2012). The test apparatus consisted of a shuttle box divided into two compartments of identical dimensions (32 cm × 15 cm × 25 cm; Varese, Italy), separated by a guillotine door (4 cm wide × 6 cm high). Each compartment featured distinct visual (e.g., wall patterns, floor patterns) and textured cues. Mice were habituated to the apparatus for 10 min per day over 3 consecutive days to reduce the influence of novelty and stress. In the pre-conditioning phase (day 1), sham or SNI 3 d animals were placed in the apparatus with the door removed, allowing them free access to both compartments for 10 min. The time spent in each compartment was recorded, and animals exhibiting a strong unconditioned preference (>400 s) were excluded from the study. During the conditioning phase (days 2–4), mice received an injection of vehicle (5 μL saline, i.th.) in the morning and were immediately confined to the paired compartment for 30 min. Approximately 6 h later, the mice were injected with lidocaine (0.04% in 5 μL saline, i.th.) and placed in the other compartment for 30 min. The chambers were counterbalanced for drug pairing across all test mice. The CPP test was conducted on day 5, during which mice were placed in the apparatus and allowed free access to both compartments for 10 min, with the time spent in each compartment recorded for each animal.

### 2.7 Drug administration

Intrathecal injection (*i.th.*) was given in a volume of 5 μL by percutaneous puncture through an intervertebral space at the level of the 5th or 6th lumbar vertebra, as described previously (He et al., 2012; Chen et al., 2019, 2023).

### 2.8 Immunostaining

Mice were anesthetized and subsequently perfused with phosphate-buffered saline (PBS) and 4% paraformaldehyde. L4 DRGs were dissected, post-fixed, dehydrated, and cryo-sectioned into 20 μm slices. After blocking for 1 h at 25°C in 0.01 M PBS containing 10% goat serum and 0.3% Triton X-100, sections from *Thy1*-GCaMP6s mice were incubated overnight at 4°C with the following primary antibodies: rabbit anti-GS (1:1000, Abcam, ab176562), mouse anti-NF200 (1:500, Sigma, N0142), biotinylated IB4 (1:100, Santa Cruz, sc-20701), mouse anti-CGRP (1:50, Abcam, ab4901), mouse anti-NeuN (1:50, GeneTex, GTX30773). After removal of excess primary antibodies, all sections were incubated for 2 h at room temperature with secondary antibodies conjugated to Alexa-647, specific to chicken, rabbit, or mouse antigens. Sections were then incubated with Neurotrace (1:200, Thermo Fisher, N21479) for 20 min and washed in PBS. After staining, apply DAPI-containing sealer (20–50 μL/section, Solarbio, S2110) on the section before coverslip. Immunofluorescence images were captured using a Leica DMI4000 fluorescence microscope with a DFC365FX camera (Leica) and analyzed using NIH ImageJ software. For each staining, we analyzed 3 sections per DRG, from 6 DRGs obtained from 3 mice per group. A cell was considered positive if the mean intensity of the soma exceeded three times the standard deviation (SD) of the extracellular space intensity.

### 2.9 Imaging data analysis

Ca^2+^ imaging data were analyzed *post hoc* using NIH ImageJ software. In cases where the animal exhibited signs of struggle during image collection, image frames from those segments were excluded from quantification. All imaging stacks were aligned through registration using NIH ImageJ plug-in StackReg. GCaMP6s was localized within the cytoplasm of DRG neurons. Regions of interest (ROIs) corresponding to visually identifiable somata were chosen for quantification, typically at a depth ranging from 30 to 200 μm below the surface of the DRG. The Δ*F*/*F*_0_ was computed as Δ*F*/*F*_0_ = (*F* – *F*_0_)/*F*_0_ × 100%, where *F*_0_ is the baseline fluorescence signal, which was averaged over a 2-s period corresponding to the lowest fluorescence signal recorded during the 2.5-min recording period. Active neurons were defined based on the criteria that Ca^2+^ transients exceeded a threshold set at 3 × SD of baseline activity.

Due to the extended decay time constant of GCaMP6s fluorescence, it is challenging to report firing rates solely based on Ca^2+^ responses. To compare spontaneous neuronal activity across different behavioral states, we calculated the average of Δ*F*/*F*_0_ over a 120-s recording period for each neuron. Neurons were considered responsive to peripheral sensory stimulus if the stimulus-evoked somatic activity exceeded 3 × SD of baseline activity. To calculate the stimuli-evoked activity, we calculated the average of Δ*F*/*F*_0_ for 10 s post-stimulus minus the average of 10 s pre-stimulus. For the same population of cells, z-projection with average intensity revealed all DRG neurons expressing GCaMP6s, and this was taken as the total population of neurons.

### 2.10 Statistics

Data were presented as mean ± SEM. Statistical analysis was conducted using Prism software (GraphPad 7.0, La Jolla, CA, USA). To assess differences between two populations, unpaired or paired *t*-tests were employed. For comparisons involving more than two groups, one-way or two-way (RM) ANOVA was applied, followed by Bonferroni’s or Tukey’s test. Significant levels were set at *p* ≤ 0.05.

## 3 Results

### 3.1 GCaMP is broadly expressed in primary sensory neurons within L4 DRG

**Figure 1:**
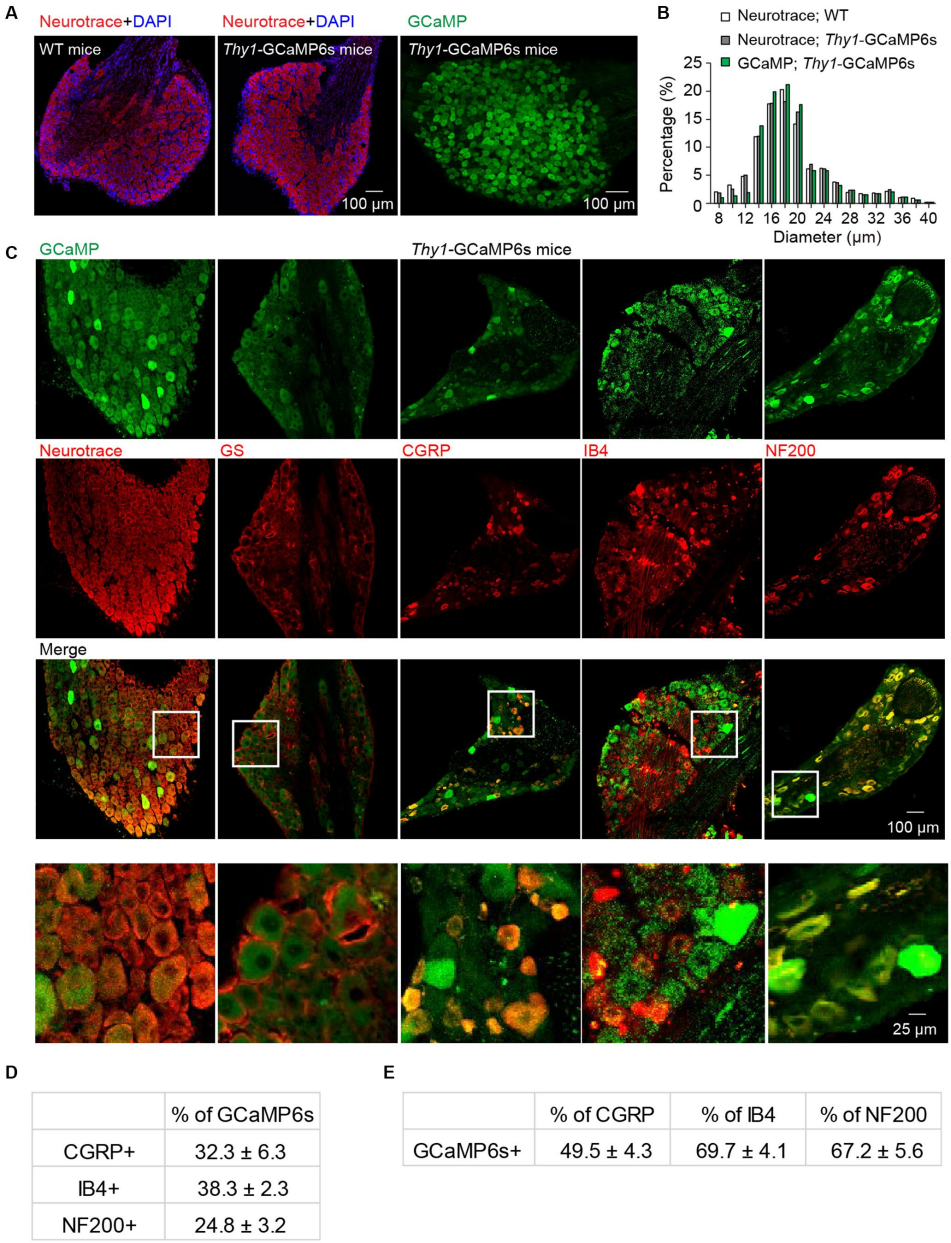
Characterization of GCaMP-expressing sensory neurons in L4 DRG of transgenic *Thy1*-GCaMP6s mice. **(A)** Immunostaining of L4 DRGs from wild-type (WT) and *Thy1*-GCaMP6s mice using Neurotrace and DAPI. **(B)** GCaMP-positive neurons display a size distribution similar to that of sensory neurons from wild-type mice. **(C)** Immunostaining of L4 DRG of *Thy1*-GCaMP6s mice with Neurotrace, GS, CGRP, IB4, and NF200. **(D)** Percentage of GCaMP6s neurons that are CGRP-, IB4-, and NF200-positive. **(E)** Percentage of CGRP, IB4 and NF200 cells that are GCaMP6s-positive.

To confirm the neuronal expression of GCaMP in the L4 DRGs of *Thy1*-GCaMP6s transgenic mice, we conducted NeuroTrace (Nissl) staining (Figure 1A), a conventional method for visualizing neurons. Analysis of GCaMP (GFP)-expressing somas revealed a widespread expression of GCaMP across small-, medium- and large-sized DRG cells. The size distribution of GCaMP-expressing cells resembled the distribution of DRG neurons in wild-type mice (Figure 1B). As expected, none of the GCaMP-expressing cells exhibited glutamate synthetase (GS) expression, a marker for satellite glial cells. Further analysis indicated that, on average, 70.3 ± 6.5% of Nissl-positive neurons expressed GCaMP (Figure 1C). Among those, 24.8 ± 3.2% of GCaMP-expressing neurons were positive for neurofilament-200 (NF200), which serves as a marker for medium and large cells as well as myelinated Aβ-fibers, while 32.3 ± 6.3% expressed calcitonin gene-related peptide (CGRP), a marker for small peptidergic neurons, and 38.3 ± 2.3% expressed isolectin B4 (IB4), a marker for small nonpeptidergic neurons (Figure 1C–E). Therefore, in *Thy1*-GCaMP6s mice, GCaMP is expressed in a representative subset of L4 DRG sensory neurons, consistent with previous reports (Chen et al., 2019).

### 3.2 Mechanical stimulus-evoked Ca^2+^ responses in DRG sensory neurons

**Figure 2:**
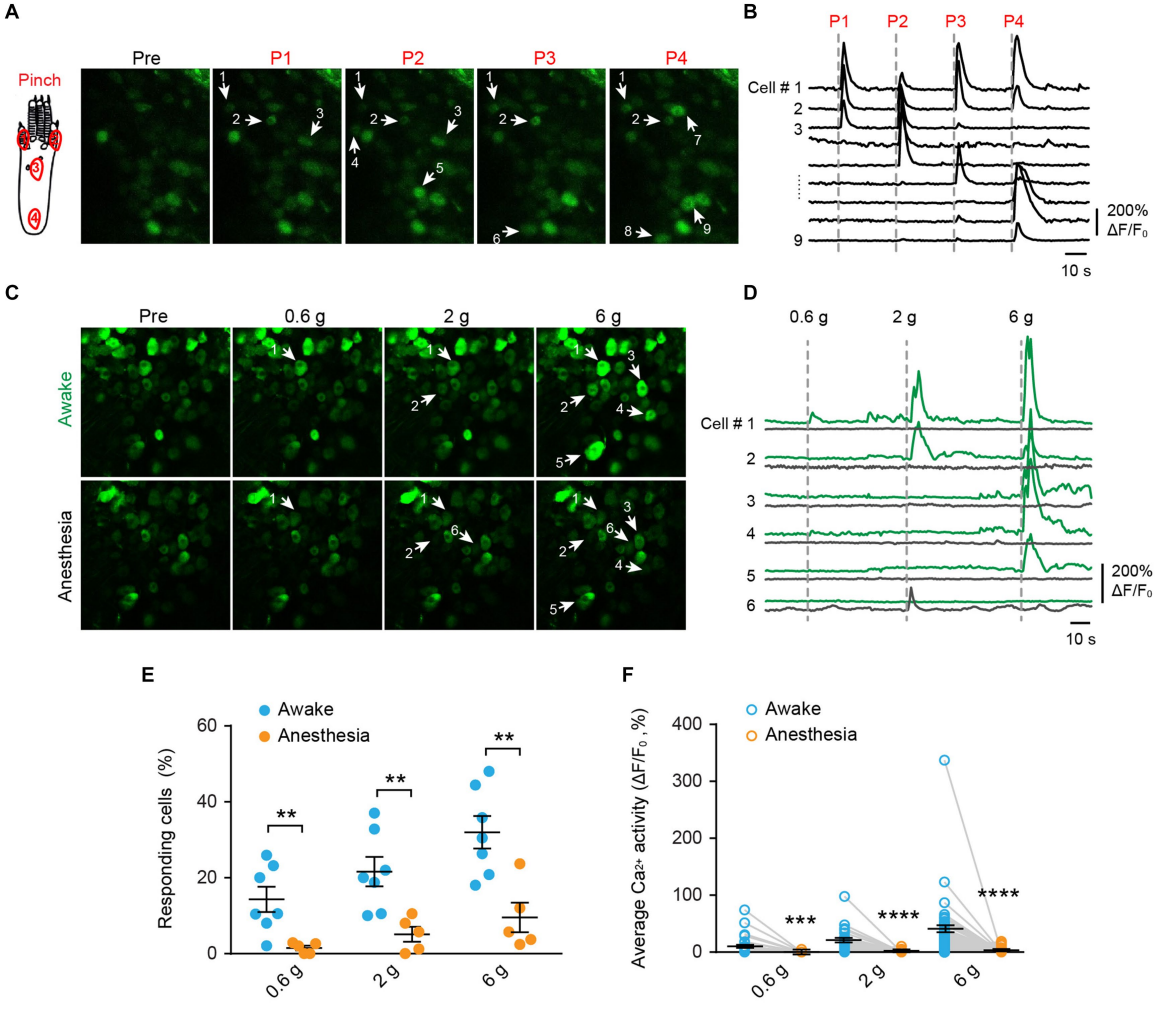
Mechanical stimulation induces Ca^2+^ responses in distinct subsets of DRG neurons in a dose-dependent manner, which is suppressed by general anesthesia. **(A,B)** Representative two-photon images and Ca^2+^ traces of L4 DRG neurons upon mechanical pinch stimulation at 4 different positions on the plantar surface of the mouse hind paw. **(C,D)** Representative images and Ca^2+^ trace of DRG neurons upon punctate mechanical stimulation (i.e., 0.6, 2, and 6 g von Frey) in awake mice (green) and anesthetized mice (black). **(E)** Percentage of DRG neurons responding to punctate mechanical stimulation in awake mice before and after anesthesia. 0.6 g: *p* = 0.0098; 2 g: *p* = 0.0075; 6 g: *p* = 0.0042. **(F)** Average Ca^2+^ activity of DRG neurons evoked by punctate stimulation in awake mice before and after anesthesia. 0.6 g: *p* = 0.0003; 2 g: *p* < 0.0001; 6 g: *p* < 0.0001. *n* = 232 neurons from five mice. Data are mean ± s.e.m. **p < 0.01, ***p < 0.001, ****p < 0.0001 versus anesthesia; by two-way RM ANOVA followed by Dunnett’s multiple comparisons test.

To investigate the mechanical modality of DRG sensory neurons, we performed *in vivo* Ca^2+^ imaging in the L4 DRG of *Thy1*-GCaMP6s mice using an implanted vertebral window (Chen et al., 2019). In awake mice, we administered pinch stimulus to the plantar surface of their hind paws, each time targeting a different site as indicated in Figure 2A. We observed that only a subset of DRG neurons responded to the pinch stimulus, and different subsets of DRG neurons responded to pinches applied to distinct sites (Figures 2A,B). These results indicate that primary sensory neurons exhibit discrete receptive fields within the paw.

When we applied a sequence of mechanical stimuli with increasing force (0.6-g, 2.0-g, 6.0-g von Frey filaments) to the same location (i.e., the sural nerve territory) with 5-min intervals (Figures 2C,D), we observed that the number of cells responding to the stimulus (i.e., responders) increased with the stimulus intensity. Out of 232 neurons recorded from 5 mice, approximately 14.3 ± 3.3%, 21.6 ± 3.9%, and 32.0 ± 4.3% of cells exhibited increased Ca^2+^ activity upon 0.6-g, 2.0-g, and 6.0-g stimulation, respectively (Figure 2E). At the individual neuron level, the magnitude of evoked Ca^2+^ responses also increased with stimulus intensity. For example, the neuronal response to 6.0-g stimulation was approximately 4 times that of the response to 0.6-g stimulation (Figure 2F).

Notably, the use of general anesthesia substantially attenuated DRG neuronal responses to mechanical stimuli (Figures 2E,F). In mice anesthetized with ketamine and xylazine, only 1.2 ± 0.5%, 4.4 ± 1.6%, and 8.0 ± 3.0% of DRG neurons responded to 0.6-g, 2-g, and 6-g stimulation, respectively. These percentages were reduced by 93.2, 76.6, and 75% compared to the awake conditions (Figure 2E and Table 1).

**Table 1:** Number of DRG neurons responsive to mechanical stimuli in awake mice before and after anesthesia.

	Total	Responsive (awake)			Responsive (anesthesia)		
		0.6 g	2 g	6 g	0.6 g	2 g	6 g
Mouse 1	50	4	5	6	1	4	6
Mouse 2	35	10	10	24	1	2	2
Mouse 3	82	19	18	25	0	1	2
Mouse 4	27	7	10	12	0	0	1
Mouse 5	38	4	4	10	1	4	9
Sum	232	44	47	80	3	11	20

### 3.3 Polymodality is a significant feature of primary sensory neurons in awake and anesthetized mice

**Figure 3:**
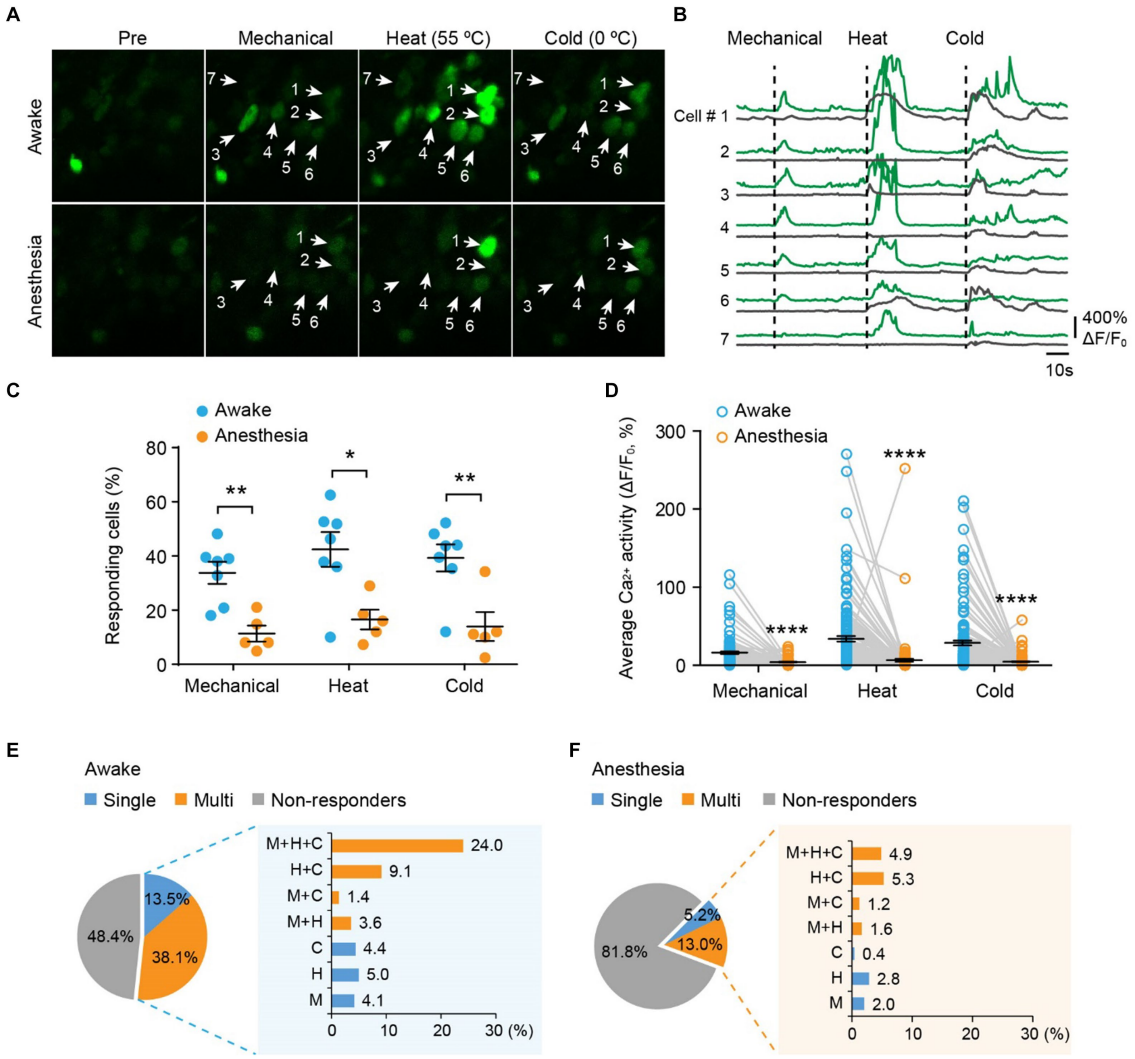
Polymodality is a prominent feature of DRG sensory neurons in both awake and anesthetized mice. **(A)** Representative two-photon images of L4 DRG neurons expressing GCaMP in response to tactile mechanical (fine brush), noxious heat (55°C), and cold (0°C) stimulation on the plantar surface of the hind paw. **(B)** Corresponding Ca^2+^ traces of neurons in **(A)**. **(C)** Percentage of DRG neurons responding to tactile mechanical (fine brush), heat (55°C), and cold (0°C) stimulation in awake mice, before and after anesthesia. Mechanical: *p* = 0.0022; heat: *p* = 0.0106; cold: *p* = 0.0068. **(D)** Average Ca^2+^ activity of responsive neurons. Mechanical: *p* < 0.0001; heat: *p* < 0.0001; cold: *p* < 0.0001. **(E,F)** Percentage of DRG neurons responsive to single or multi-modality stimulations under awake **(E)** and anesthesia **(F)** conditions. M, tactile mechanical; H, heat; C, cold. *n* = 362 neurons from seven mice. Data are mean ± s.e.m. *p < 0.05, **p < 0.01, ****p < 0.0001 versus anesthesia; by two-way RM ANOVA followed by Dunnett’s multiple comparisons test.

Next, we examined the responses of DRG sensory neurons to different stimulus modalities. We sequentially applied mechanical (light brush), cold (0°C water), and heat (55°C water) stimuli to the same area of the plantar surface to stimulate neurons with the same receptive field (Figures 3A,B). In awake mice, we observed that an average of 33.8 ± 4.1% of neurons responded to mechanical stimuli, 42.4 ± 6.4% responded to heat, and 39.3 ± 5.0% responded to cold (Figure 3C). Among all responders, 72.7, 59.1, and 65.9% of them became non-responsive after ketamine-xylazine anesthesia (Figure 3C). Furthermore, stimuli-evoked Ca^2+^ activity was significantly attenuated by anesthesia (Mech: 13.5 ± 1.5 vs. 1.5 ± 0.36; Heat: 31.4 ± 3.5 vs. 3.8 ± 1.8; Cold: 26.1 ± 3.1 vs. 1.9 ± 0.55; awake versus anesthesia, *p* < 0.0001; Figure 3D).

Among all neurons assessed (100%), including responsive and non-responsive, we found that 13.5% were modality specific. This category included 4.1% mechanical-specific neurons, 5.0% heat-specific neurons, and 4.4% cold-specific neurons (Figure 3E). Notably, a larger fraction (38.1%) of sensory neurons demonstrated responsiveness to two or three sensory modalities. Specifically, 3.6% of neurons were responsive to mechanical and heat, 1.4% were responsive to mechanical and cold, 9.1% were responsive to heat and cold, and 24.0% were responsive to all three stimuli (Figure 3E). Although ketamine-xylazine anesthesia reduced the number of responders to 18.2% among the neurons assessed, the majority of remaining responders remained polymodal — 5.2% of all neurons responsive to single stimuli and 13.0% responsive to multiple stimuli (Figure 3F). Collectively, our data indicate that polymodality is a prominent feature of DRG sensory neurons, regardless of whether the animal is awake or under anesthesia.

### 3.4 Peripheral nerve injury increases the spontaneous activity of DRG neurons

**Figure 4:**
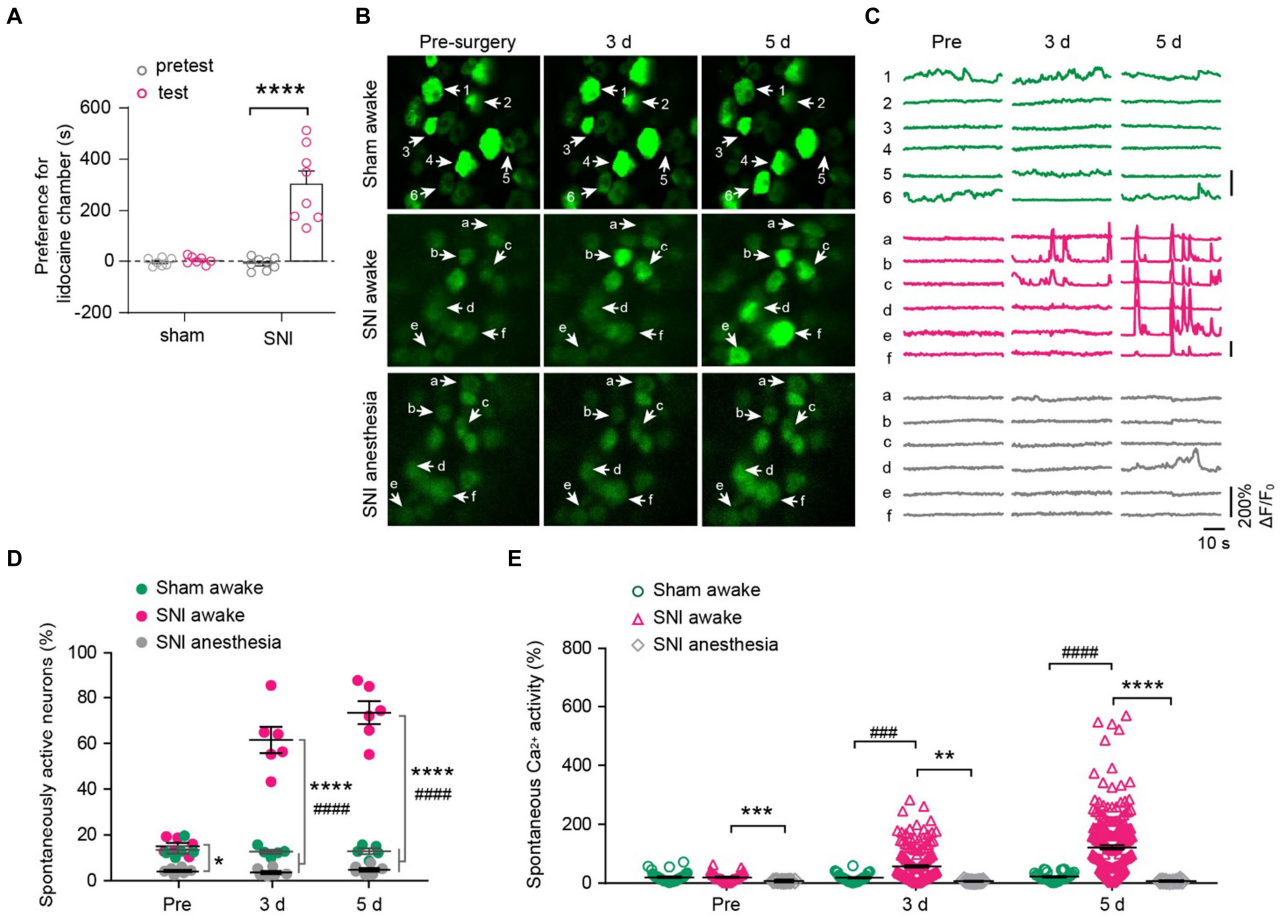
DRG neurons exhibit increased spontaneous activity after spared nerve injury (SNI) and become silent during anesthesia. **(A)** The conditioned place preference (CPP) test. Time spent in lidocaine paired compartments minus that in saline paired compartments are shown for sham and SNI mice. *p* < 0.0001. *n* = 7–8 mice per group. **(B,C)** Representative two-photon images and Ca^2+^ traces of L4 DRG neurons that are spontaneously active before (pre) and 3–5 days after SNI. **(D)** Percentage of spontaneously active DRG neurons in sham and SNI mice with or without anesthesia. SNI awake vs. sham awake, Pre: *p* = 0.6944, 3 d: *p* < 0.0001, 5 d: *p* < 0.0001; SNI awake vs. SNI anesthesia, Pre: *p* = 0.0178, 3 d: *p* < 0.0001, 5 d: *p* < 0.0001. **(E)** Average Ca^2+^ activity of spontaneously active neurons in L4 DRG of mice with or without anesthesia. SNI awake vs. sham awake, Pre: *p* = 0.9996, 3 d: *p* = 0.0002, 5 d: *p* < 0.0001; SNI awake vs. SNI anesthesia, Pre: *p* = 0.0001, 3 d: *p* = 0.0011, 5 d: *p* < 0.0001. *n* = 274 neurons from 5 mice in sham, *n* = 299 neurons from 6 mice in SNI group. Data are mean ± s.e.m. *p < 0.05, **p < 0.01, ***p < 0.001, ****p < 0.0001, SNI awake versus SNI anesthesia; ^###^*p* < 0.001, ^####^*p* < 0.0001, SNI awake versus sham awake; by paired *t*-test **(A)** or two-way ANOVA followed by Dunnett’s multiple comparisons test **(D,E)**.

To determine the impact of peripheral neuropathic pain on the sensory modalities of DRG neurons, we used a spared nerve injury (SNI) model to induce peripheral neuropathic pain in mice. After undergoing either sham or SNI surgery, mice were subjected to a two-compartment conditioned place preference (CPP) test. The results showed that SNI mice spent more time in the compartment paired with analgesic lidocaine compared to the saline-paired compartment, whereas sham-operated mice spent a similar amount of time in both compartments (time in the lidocaine-paired chamber: 451.6 ± 25.6 s for SNI versus 302.2 ± 2.4 s for sham; *p* < 0.0001) (Figure 4A). These results confirmed the development of spontaneous ongoing pain in SNI mice.

We then examined how peripheral nerve injury affects the spontaneous activity of DRG sensory neurons. In line with previous reports (Chen et al., 2019), we observed that a small fraction of sensory neurons (~14%) in the L4 DRG of naïve mice exhibited spontaneous Ca^2+^ activity under resting awake conditions (Figures 4B–D). This population of spontaneously active cells remained stable over days after sham surgery (pre-sham, 13.5 ± 1.6%, 3d, 12.7 ± 0.9%; 5d, 12.9 ± 1.2%). However, it increased enormously after SNI (pre-SNI, 15.1 ± 1.4%, 3d, 61.5 ± 5.7%; 5d, 73.5 ± 5.0%) (Figures 4C,D). At the individual cell level, the average somatic Ca^2+^ activity remained stable over days in sham-operated mice (Pre, 6.9 ± 1.3; 3d, 5.2 ± 1.0; 5d, 6.2 ± 1.1). In contrast, the somatic activity of DRG neurons increased tremendously by 5.1-fold 5 days after SNI (Pre, 4.4 ± 0.7; 3d, 59.2 ± 4.1; 5d, 152.8 ± 7.1) (Figure 4E). Similar to what was observed in naïve mice (Figure 2), anesthesia reduced the population of spontaneously active neurons in SNI mice (Pre, 4.3 ± 0.5%; 3d, 3.6 ± 0.7%; 5d, 4.8 ± 0.8%; Figure 4D). Anesthesia also largely abolished the Ca^2+^ activity of individual DRG neurons in SNI mice (Figure 4E).

### 3.5 Peripheral nerve injury induces the generalization of sensory modalities

**Figure 5:**
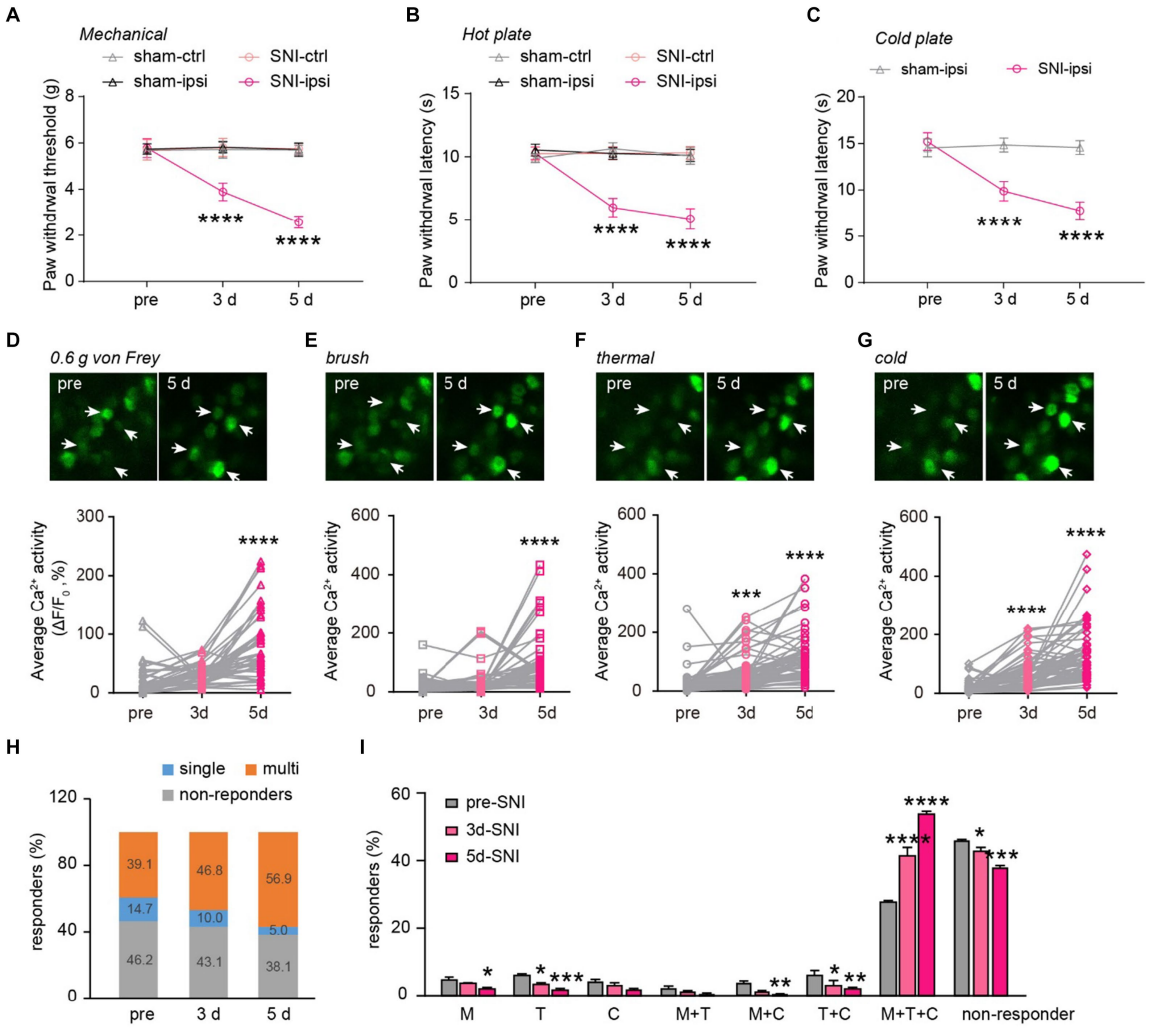
Generalization of sensory modality responses in neuropathic pain. **(A)** Paw withdrawal thresholds to von Frey mechanical stimulation before (pre) and 3–5 days after sham or SNI surgery. **(B,C)** Paw withdrawal latencies to noxious heat (55°C) and cold (0°C) before and 3–5 days after sham or SNI surgery. *n* = 7–8 mice per group. **(D–G)** Upper, representative Ca^2+^ images of DRG neurons upon punctate mechanical (0.6 g von Frey), tactile mechanical (brush), heat (55°C), and cold (0°C) stimulation. Lower, stimulation evoked Ca^2+^ activity in mice before and 3–5 days after SNI. 0.6 g, 3d:*p* = 0.3736; 5d: *p* < 0.0001. Brush, 3d:*p* = 0.6210; 5d: *p* < 0.0001. Thermal, 3d: *p* = 0.0009; 5d: *p* < 0.0001. Cold, 3d: *p* < 0.0001; 5d: *p* < 0.0001. **(H)** Percentage of DRG neurons that are non-responsive or responsive to single or multiple sensory modalities. **(I)** Percentage of DRG neurons that are non-responsive or responsive to different sensory stimulation. M, 3d: *p* = 0.5603, 5d: *p* = 0.0231; T, 3d: *p* = 0.0356, 5d: ***p = 0.0002; C, 3d: *p* = 0.8894, 5d: *p* = 0.0662; M + T, 3d: *p* = 0.8205, 5d: *p* = 0.3356; M + C, 3d: *p* = 0.0597, 5d: *p* = 0.0097; T + C, 3d: *p* = 0.0136, 5d: *p* = 0.0012; M + T + C, 3d: *p* < 0.0001, 5d: *p* < 0.0001; non-active, 3d: *p* = 0.0249, 5d: *p* < 0.0001; *n* = 299 neurons from 6 mice. Data are mean ± s.e.m. ****p < 0.0001 versus sham-ipsi **(A–C)**; *p < 0.05, **p < 0.01, ***p < 0.001, ****p < 0.0001 versus pre-SNI **(D–G,I)**; by two-way RM ANOVA followed by Dunnett’s multiple comparisons test **(A–C,I)** or one-way RM ANOVA followed by Dunnett’s multiple comparisons test **(D–G)**.

Next, we explored how peripheral nerve injury affects stimulus-evoked activity in DRG sensory neurons and their sensory modalities. Behaviorally, SNI mice displayed mechanical and thermal allodynia on the ipsilateral paw 3 and 5 days after surgery (Figures 5A–C). This was evident in their reduced paw withdrawal thresholds to mechanical stimuli (SNI-ipsi versus sham-ipsi: 3d, 3.9 ± 0.4 versus 5.8 ± 0.2; 5d, 2.6 ± 0.3 versus 5.7 ± 0.3) and withdrawal latency to heat (3d: 6.0 ± 0.3 versus 10.3 ± 0.2; 5d: 5.1 ± 0.3 versus 10.1 ± 0.2) and cold stimuli (3d: 9.9 ± 0.4 versus 14.5 ± 0.3 s; 5d: 7.8 ± 0.4 versus 14.6 ± 0.3). No significant differences were observed in nociceptive thresholds for the contralateral paw between SNI and sham mice (Mechanical 3d: 5.8 ± 0.4 g versus 5.7 ± 0.4; 5d: 5.7 ± 0.2 versus 5.7 ± 0.2. Thermal 3d: 10.3 ± 0.2 s versus 10.6 ± 0.2; 5d: 10.3 ± 0.2 versus 10.1 ± 0.2 s) (Figures 5A–C).

When we measured the level of Ca^2+^ activity in individual DRG neurons, we found that a non-noxious mechanical stimulus (i.e., 0.6-g von Frey) elicited significantly higher spiking activity in DRG neurons of SNI mice compared to the pre-SNI baseline (Figure 5D). A similar trend was also observed in the level of evoked Ca^2+^ activity in response to the mechanical brush, heat, and cold stimuli before and after SNI (Figures 5E–G). Furthermore, we observed a larger population of DRG neurons becoming responsive to mechanical, cold, or heat stimuli 3 and 5 days after SNI. Importantly, the modality-specific population decreased progressively, from 14.7% of total neurons before SNI to 5.0% on day 5, while the polymodal population increased from 39.1% at baseline to 56.9% on day 5 after SNI (Figures 5H,I). These results indicate that peripheral neuropathy generalizes the sensory modality responses of DRG neurons.

## 4 Discussion

Using *in vivo* Ca^2+^ imaging of DRG neurons in awake mice, we found that a significant portion of somatosensory neurons responded to multiple sensory stimuli (i.e., polymodal). Anesthesia substantially reduced the number of responsive neurons but had no impact on their polymodal characteristics. Furthermore, the polymodality feature of DRG sensory neurons was further enhanced after peripheral nerve injury.

Primary somatosensory neurons exhibit spatially distinct receptive fields (Ma et al., 2003; Zhu and Henry, 2012; Queme et al., 2022). To validate our experimental setup, we repeatedly applied the same mechanical stimulus to the same or different receptive fields of the mouse’s hind paw. We found that a 2-g von Frey stimulus applied to the same receptive field consistently activated the same subpopulation of neurons in a reproducible manner. In contrast, when the same stimulus was applied to distant receptive fields, it activated distinct groups of neurons with minimal overlap. These results validate the reliability of our experimental design in detecting peripheral sensory stimulus-evoked responses of primary sensory neurons. Based on these findings, we subsequently applied different sensory stimuli to the same receptive field and assessed the modality responses of DRG neurons.

Recent *in vivo* imaging studies have presented contradicting views on sensory neuron modality. Emery et al. reported polymodality as a relatively infrequent feature of sensory neurons. In their study, out of 17 GCaMP6s-expressing cells sampled, only 3 were polymodal, accounting for ~15% of the neurons (Emery et al., 2016). In contrast, Chisholm et al. demonstrated polymodality as a primary feature of DRG neurons. Among 1,138 neurons sampled, ~55% of temperature-responsive neurons were also responsive to pinch (Chisholm et al., 2018). The smaller sampling size in Emery et al. study may partially explain the discrepancy, and differences in their methods of calculating polymodality could further contribute to the inconsistency. Emery et al. calculated the percentage of thermally responsive neurons per mechanically responsive cells, while Chisholm et al. calculated the ratio of mechanically responsive neurons per thermally responsive cells. In the present study, we sampled a relatively large number of DRG neurons (*n* = 362 neurons from 7 mice) and calculated the percentage of responsive neurons among all neurons identified. Our data provide support for polymodality as a significant feature of primary sensory neurons in the DRG, regardless of whether the mouse is awake or anesthetized.

The technology for accessing large numbers of primary afferents *in vivo* under awake conditions has become available only more recently (Weber et al., 2006; Chen et al., 2019; Sun et al., 2022; Chen et al., 2023; Zhou et al., 2023). Before this development, general anesthesia, administered through inhalation or injection of various anesthetics/sedatives, was extensively used to enable *in vivo* access to mouse DRGs (Arras et al., 2001; Emery et al., 2016; Kim et al., 2016; Smith-Edwards et al., 2016; Chisholm et al., 2018). It is important to note that different anesthetic cocktails may affect peripheral sensory neurons in distinct ways. For instance, ketamine alters the activation and inactivation properties of tetrodotoxin-sensitive and -resistant sodium channels in primary sensory neurons (Zhou and Zhao, 2000), while isoflurane inhibits excitatory transmitter release by blocking presynaptic Ca^2+^ channels and exocytic machinery (Wang et al., 2020). Variations in anesthesia regimes, such as the choice of drugs and dosages, may have contributed to the differing conclusions in previous studies (Emery et al., 2016; Chisholm et al., 2018). General anesthetics, known as potent modulators of neuronal activity, induce loss of sensation and relief of pain (Huang et al., 2016), which can confound the physiological assessments of somatosensory neurons. Indeed, our results demonstrated that systemic administration of ketamine-xylazine reduced the number of DRG cells responsive to peripheral sensory stimuli by 72.7% in mechanical, 59.1% in heat and 65.9% in cold, respectively (Figure 3C). Among DRG neurons that remained active during anesthesia, the level of stimulus-evoked activity decreased by 89.0% in mechanical, 87.8% in heat and 92.6% in cold, respectively. (Figure 3D). Similarly, in mice with neuropathic pain, ketamine-xylazine anesthesia mostly abolished the spontaneous activity of DRG neurons (Figure 4E). In addition, the generation of ganglia-specific fluorescence-expressing transgenic mice has significantly facilitated the functional research in the pain field (Taylor-Clark et al., 2015; Miller et al., 2018; Chen et al., 2019; Cichon et al., 2020; Wang et al., 2022). Many factors, including transcription, epigenetic regulators, cellular components, can affect the transgenic expression patterns of proteins under the same promoter, here *Thy1.2*. In our previous study, we reported differences in both the expression pattern and level of GCaMP in neurons across *Thy1.2*-GCaMP6 slow lines 1 and 3, as well as fast lines (Cichon et al., 2020). We also employed *Thy1.2*-YFP mouse line for structural imaging of DRG (Chen et al., 2019), which labels fewer neurons, with the majority of YFP-positive neurons being medium to large in diameter, as previously reported (Taylor-Clark et al., 2015). Therefore, it’s of great importance to choose the appropriate labeling method for DRG imaging study.

Phenotypical shifts in DRG neurons occur in response to peripheral nerve injury or insults, including changes in molecular expressions (Wang et al., 2011), cellular excitability (Zhao et al., 2017), and evoked neuronal responses (Kim et al., 2016). In this study, we report that peripheral nerve injury activates a large population of previously quiescent sensory neurons, with over 60% of DRG neurons exhibiting spontaneous Ca^2+^ transients compared to ~15.1% before surgery (Figure 4D). This finding aligns with previous reports indicating that a subset of primary sensory neurons exhibits lower resting membrane potentials and heightened neuronal excitability due to reduced expression of voltage-gated potassium channels, such as Kv1.2 (Zhao et al., 2013, 2017). Furthermore, peripheral injury or inflammation has been shown to reveal silent nociceptors and even alter neuronal responses to different sensory modalities (Perl, 1996; Emery et al., 2016; Chisholm et al., 2018). In our study, we conducted a longitudinal observation on the same population of DRG sensory neurons, subjecting them to various sensory stimuli before and after peripheral nerve injury. We observed that innocuous punctate mechanical stimulation (0.6-g von Frey) elicited greater Ca^2+^ activity after SNI, potentially contributing to SNI-induced mechanical allodynia. Similarly, noxious mechanical, cold, and heat stimuli induced higher activity levels in DRG neurons after SNI. Analysis of modality responses before and after SNI indicated that polymodality remains a central feature of DRG neurons following peripheral nerve injury, and the proportion of polymodal neurons increases over the course of days after SNI. These changes in the sensory modalities of DRG neurons stem from SNI-induced molecular changes, as previous studies have reported that SNI can activate the expression of TRPV1 in neurons that were previously TRPV1-negative (Wang et al., 2011).

Of note, although GCaMP6s produced large fluorescence transients due to somatic calcium increase triggered by a single action potential (Chen et al., 2013), its slow dynamics makes it difficult to resolve the number of action potentials. Therefore, our *in vivo* imaging data were not able to decode the firing rates of these neurons. The mice were allowed 5–7 days for recovery from window implantation before the *in vivo* DRG imaging. However, since the invasive surgical procedures of DRG window implantation caused increased macrophage infiltration (Chen et al., 2019), the wake activities we recorded might not fully represent the “normal” state of these neurons. Repeated imaging could be performed as long as the window is clear, and we could succeed 90% of the time for repeated imaging within a week. Therefore, we focused on the developmental phase of neuropathic pain - the initial 5 days post nerve injury. For the modality-related response, the water droplet stimulations used in the study could also produce mechanical force on the hindpaw. However, given that mechanical alone neurons could still be identified in Figures 3, 5, and the mech + heat, mech + cold population did not encompass all mechanical-responsive neurons, we believe that the method allowed for differentiation of modality-related response between sham vs. SNI as well as awake vs. anesthesia situations. Given that our study exclusively examined DRG neurons from male mice, employed water droplets for hot and cold stimulations, and focused on the early phase of neuropathic pain, it is crucial to interpret the results with caution.

In summary, through the use of an awake animal DRG imaging technique, our study identifies polymodality as a significant feature of DRG sensory neurons, which is further enhanced in the context of peripheral neuropathic pain.

## Data availability statement

The original contributions presented in the study are included in the article, further inquiries can be directed to the corresponding authors.

## Ethics statement

The animal study was approved by Institutional Animal Care and Use Committee (IACUC) at New York University and Columbia University. The study was conducted in accordance with the local legislation and institutional requirements.

## Author contributions

LS: Writing – original draft, Writing – review & editing. CC: Writing – original draft, Writing – review & editing. XX: Writing – original draft, Writing – review & editing. SG: Writing – original draft, Writing – review & editing. GY: Writing – original draft, Writing – review & editing.
